# Intervention for reducing anxiety during screening mammography: A protocol for systematic review and meta-analysis

**DOI:** 10.1097/MD.0000000000022382

**Published:** 2020-10-09

**Authors:** Yi Shang, Zi-Wei Song, Li Du, Li-Ping Yang, Zhi-Gang Zhang

**Affiliations:** a Department of General Surgery, Lanzhou University Second Affiliated Hospital; b Evidence-Based Nursing Center, School of Nursing, Lanzhou University; c Department of Psychiatry, Lanzhou University Second Affiliated Hospital, Lanzhou, Gansu Province, China; d Department of General Surgery, The First Hospital of Lanzhou University; e School of Nursing, Lanzhou University; f Intensive Care Unit, The First Hospital of Lanzhou University, Lanzhou, China.

**Keywords:** anxiety, mammography, screening

## Abstract

**Background:**

Mammography is considered a fundamental part of diagnosis in modern health care services. It provides low dose images of normal structures and pathological soft tissues in the breast. Many reports suggested that intervention is playing a positive role in anxiety related to mammography, but there is no high-quality evidence to prove its effects. This paper reports the protocol of a systematic review (SR) and meta-analysis (MA) to clarify effectiveness of intervention during screening mammography.

**Methods:**

A systematic literature search will be performed in the Cochrane Library, PubMed, Embase and Web of Science from inception to July 2020. Randomized controlled trials (RCTs) will be included to evaluate any interventions in the treatment of anxiety related to mammography screening. The main outcome measure is the impact on patient anxiety, and the impact on patient breast cancer worry, the impact on patient satisfaction are the additional outcome measure. Risk of bias assessment of the included RCTs will be carried out using Cochrane Collaboration's tool for RCTs. The Review Manager 5.4 for Windows will be used to perform the MA and generate the result figures. The Grading of Recommendations Assessment, Development and Evaluation (GRADE) will be used to evaluate the quality of evidence. Subgroup analysis and sensitivity analysis will be conducted to assess the robustness of the results.

**Results:**

A total of 782 English studies of anxiety related to mammography screening were obtained through search. After preliminary screening, 773 non-conforming studies were excluded. Finally, nine English studies of anxiety related to mammography screening will be included for full-text assessment. We will submit the results of this SR and MA to a peer-reviewed journal for publication.

**Conclusions:**

This study will provide reliable evidence for intervention for reducing anxiety in women receiving screening mammography.

**INPLASY registration number:**

INPLASY202070131.

## 1 Introduction

The GLOBOCAN 2018 statistical estimate of the international agency for research on cancer shows, among females, breast cancer is the most frequently diagnosed cancer and the main cause of cancer death.^[1]^ Because of the sheer magnitude of this disease, its psychosocial impact and associated morbidity and mortality, screening for early diagnosis may be one of the best strategies we can employ to fight against this insidious ailment. Mammography is considered an important part of diagnosis in modern health care services. It provides low dose images of normal structures and pathological soft tissues in the breast. For asymptomatic and symptomatic women with obvious lumps and any other discomforts, mammograms can be performed. It is a suitable source of early detection for breast cancer in diagnostic images.^[2]^ As many guidelines have already published, mammography screening can reduce the mortality of breast cancer by detecting early tumors.^[3]^ Therefore, Mammography has been proven to be the “Gold Standard” technique for breast cancer screening.^[2]^ However, anxiety can reduce adherence, and improve breast cancer mortality and morbidity. It is generally considered a harm of mammography with few options offered to reduce women's anxiety related to a mammography have been tested, their results are diverse.^[4]^ Two interventions based on relaxing music or an online support system using the comprehensive health enhancement support system^[13]^ have had a negative impact on reducing anxiety. Two interventions based on psychoeducational session^[15]^ or a protocolized nursing intervention^[14]^ have had a positive impact on reducing anxiety. There have been many RCTs that have explored the effectiveness of intervention for reducing anxiety during screening mammography, but there are a wide variety of interventions involved and the quality of RCTs is also jagged.

In order to better provide evidence for the practice of evidence-based medicine, we conducted a MA in order to screen out the best evidence of intervention for reducing anxiety during screening mammography.

## 2 Methods

### 2.1 Study registration

This protocol refers to the Preferred Reporting Items for Systematic Reviews and Meta-Analyses Protocols (PRISMA-P)^[5]^ checklist and it was registered in the International Platform of Registered Systematic Review and Meta-analysis Protocols (INPLASY) database (protocol number: INPLASY 202070131).

### 2.2 Eligibility criteria

#### 2.2.1 Type of study

RCTs that explored the effectiveness of intervention for reducing anxiety during screening mammography will be approved.

#### 2.2.2 Type of patients

Women could be of ethnic origin and patients should undergo screening mammography; women without: a current psychiatric diagnosis; a history of breast cancer or DICS (Ductal carcinoma in situ); current psychiatric treatment in any form.

#### 2.2.3 Type of interventions

Intervention measures: psychological intervention, behavioral intervention, psychological behavior intervention, medicine treatment, or any intervention combined therapy of the above four type of intervention. The control group will be no-treatment, conventional treatment or nursing.

#### 2.2.4 Type of outcomes

The primary outcome will include impact on patient anxiety, the impact on patient anxiety will be measured by Spielberger State Trait Anxiety Inventory (STAI). The intervention group and the control group should use the State Anxiety (SA) subscale of STAI to measure SA before and after mammography.^[6]^ Items will be scored on a Likert-type scale from 0 to 3. The score is calculated as the sum of the items, and the possible range is 0 to 60. The higher the score, the greater the SA. The secondary outcomes will include impact on patient breast cancer worry, impact on patient satisfaction. The Breast Cancer Worry subscale of the Lerman Breast Cancer Worry Scale (LBCWS),^[7]^ designed specifically to assess the constructs of breast cancer worry, was also administered before and after mammography in both intervention and control groups. This single item, “How worried are you about getting breast cancer someday?” will be rated on a 0 (“Not at all”) to 4 (“Almost all the time”) Likert scale. The Patient Satisfaction with Doctor Questionnaire (PSQ-MD) will be administered before and after mammography in both intervention and control groups. This scale has two subscales: Perceived Support and Physician Disengagement, in which the item's rating scale is 0 to 3, and the score calculation range is 0 to 72.^[8]^

#### 2.2.5 Exclusion criteria

Literatures or duplicate data published repeatedly by the same author; less than 10 samples in the experimental group or control group. If the type of study is protocol, review, letter comments, they will be excluded. Anxiety combined with depression or other psychological problems will be excluded.

### 2.3 Data source and search strategy

The following electronic databases have been used by two independent reviewers: Embase, Cochrane Library, PubMed, Web of Science, WHO Trials Registry, and Clinical Trials. Reference lists of articles, and grey literature will also be searched. The Language of the publication have been limited to English. The search strategy has been adapted to each database, the search terms include “Hypervigilance,” “Nervousness,” “Anxiety,” “Mammography,” “Cancer Early Detection,” and others. There was no restriction on the year of publication. The detailed search strategy is given in Tables 1 and 2.

**Table 1:**
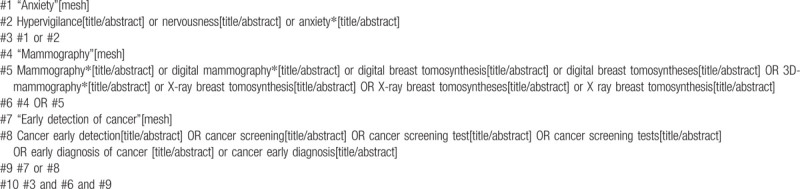
Search strategy in PubMed.

**Table 2:**
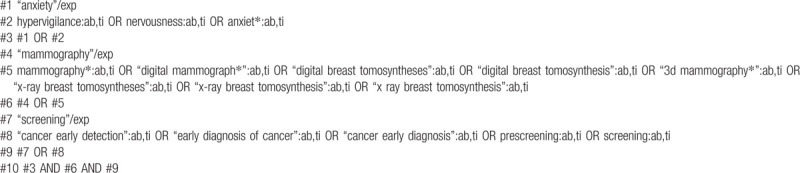
Search strategy in the Embase.

### 2.4 Study selection

We will use Endnote X9.3 to manage the search results and perform screening, according to the inclusion and exclusion criteria, two reviewers will scan the articles independently and assess the possible eligible articles as full text. If there is a disagreement between the reviewers, a third expert or all members of the group will participate in the discussion.

### 2.5 Data extraction

Two main reviewers will collect data independently on the characteristics of the studies (including the first author, year, age, education, personal health history, family history of breast cancer, and previous abnormal screening mammography, intervention (observation group, control group), and outcomes, number of trials/patients, effect size, 95% CI) using a standardized data extraction form for included trials. Disagreements will be resolved by discussion.

### 2.6 Risk of bias of individual studies

The quality of selected studies will be assessed independently by two reviewers according to the Cochrane Collaboration's tool for RCTs.^[9]^ Items will be evaluated in three categories: high risk of bias, unclear bias and Low risk of bias. The following characteristics will be evaluated: allocation concealment (selection bias), random sequence generation (selection bias), blinding of participants and personnel (performance bias), selective reporting (reporting bias), incomplete outcome data (attrition bias). other biases. Results from these questions will be graphed and evaluated using RevMan 5.4.

### 2.7 Statistical analysis

#### 2.7.1 Meta-analysis

Risk ratio (RR) for both fixed and random effects models (weighting by inverse of variance) will be used. We will assess the between-study heterogeneity using the *I*^2^ statistics. According to the Cochrane handbook, if the *I*^2^ ≤ 50%, it suggests that there is no statistical heterogeneity. We will assess the results using forest plots and presented as RRs for the main outcome and second outcomes. We will conduct statistical analysis using the statistical package (RevMan v5.4).

#### 2.7.2 Subgroup analysis and sensitivity analysis

If there is significant heterogeneity in the included trials, subgroup analysis will be performed. According to subject characteristics (eg, severity of anxiety, age, education, and so on), subgroup analysis will be carried out according to the data retrieved.

If possible, we will do some extra subgroup analysis according to the results of heterogeneity. If the evidence is sufficient, we will conduct sensitivity analysis. If probably, trials, where missing data have been imputed and high risk of bias rating have been assessed will be excluded. We will also investigate the sources of heterogeneity to determine the robustness and reliability of the consolidated results, it will be performed by deleting each study at a time, and other studies will be analyzed to assess whether a single study would have a significant impact on the results.

### 2.8 Quality of evidence

The GRADE will be used to assess the quality of evidence, It is categorized into four levels: high level, moderate level, low level, and very low level.^[10]^ GRADE takes the limitations of the studies in terms of their conduct and analysis; the directness (or applicability and external validity) of the evidence with respect to the populations, interventions and settings where the proposed intervention may be used; the consistency of the results across the available studies; the study design and the precision of the summary estimate of the effect into consideration.^[11,12]^

### 2.9 Summary of findings

For the SR and MA, we will produce “summary of findings” tables, Experimental group vs control group. In addition, we will evaluate the quality of all subgroup analyses conducted and explain reasons for downgrading. We will include the following three outcomes: patient anxiety, patient worry, patient satisfaction, the details of summary of findings are summarized in Table 3.

**Table 3:**
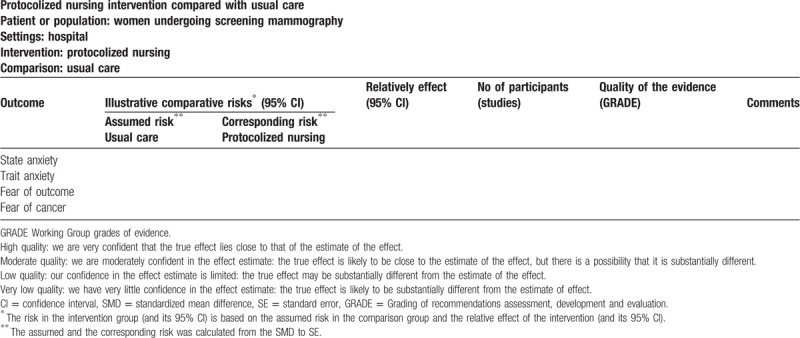
Partial summary of findings for the main comparison.

## 3 Results

### 3.1 Results of the search

**Figure 1:**
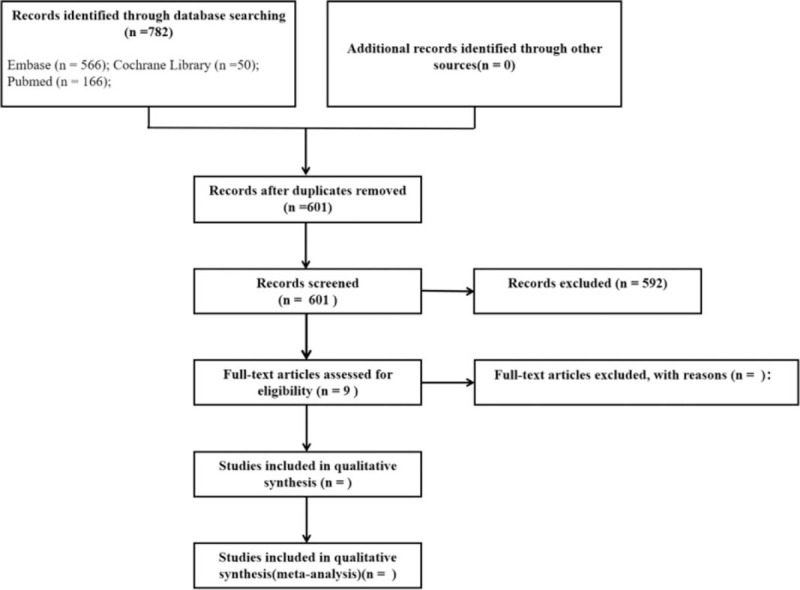
The flowchart of the screening process.

A total of 782 English studies of anxiety related to mammography screening were obtained through search. After preliminary screening, 773 non-conforming studies were excluded. Finally, nine English studies of anxiety related to mammography screening will be included for full-text assessment. The detailed search flow chart is shown in Figure 1.

### 3.2 Characteristic of included studies

We extracted the basic characteristics of some of the included studies, we conducted a preliminary experiment and included three RCTs, the minimum sample size is 50 and the maximum is 436, the age ranged from 40 to 69 years, the details of characteristics of the included studies are summarized in Table 4.

**Table 4:**
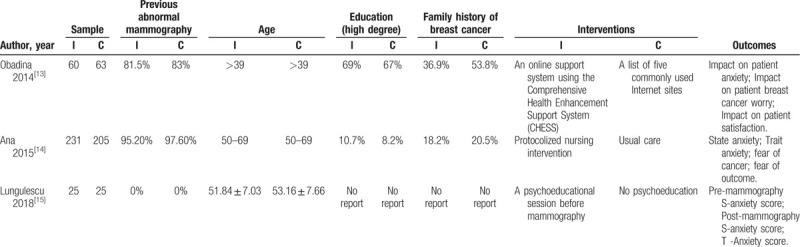
Characteristics of partial included studies.

## 4 Discussion

As far as we know, this is the first protocol for MA and SR that compares the effects of different interventions on anxiety related to mammography. The MA will be used to summarize the evidence.

We will highlight the strengths and limitations during identifying evidence. Two researchers will complete the data extraction and risk of bias assessment independently, which will provide accurate evidence for interventions for reducing anxiety. Limitations will mainly originate from different clinical situation and different basic treatment on women undergoing screening mammography. It may lead to high degree of heterogeneity and reduce the quality of the evidence. However, we will use subgroup analysis and sensitivity analysis to overcome these heterogeneities in the MA. We hope that this study can screen the best interventions to provide strong and reliable evidence of treatment for people experiencing anxiety related to mammography screening and provide recommendations for clinical practice or guidelines which assist in making choices between different interventions that have an impact on public health and resources, and help health care providers and recipients and other stakeholders to make informed decisions.^[16]^^,^^[17]^

## Author contributions

ZGZ, LPY, ZWS, and YS conceived this study. ZGZ, YS, and ZWS designed the inclusion/exclusion criteria and the searching strategy. LD designed a data extraction table. ZWS and YS will search for the literature. ZWS, YS, and LD will collect the data and made statistical analysis. ZWS, YS, LPY, and ZGZ drafted the protocol and revised the manuscript.

## Author contributions

**Conceptualization:** Yi Shang, Zi-Wei Song, Li-Ping Yang, ZhiGang Zhang.

**Methodology:** Yi Shang, Zi-Wei Song, ZhiGang Zhang.

**Writing - Original Draft:** Yi Shang, Zi-Wei Song, Li-Ping Yang, ZhiGang Zhang.

**Writing - Review & Editing:** Yi Shang, Zi-Wei Song, Li-Ping Yang, ZhiGang Zhang.

**Formal analysis:** Zi-Wei Song, Li Du.

**Data curation:** Li Du.

## Abbreviations

Abbreviations: GRADE = grading of recommendations assessment, development and evaluation, MA = meta-analysis, PRISMA = preferred reporting items for systematic review and meta-analysis, RCT = randomized controlled trial, SA = state anxiety, SR = systematic review, STAI = spielberger state trait anxiety inventory.
